# Magnesium Homeostasis in Cardiac Myocytes of Mg-Deficient Rats

**DOI:** 10.1371/journal.pone.0073171

**Published:** 2013-09-09

**Authors:** Michiko Tashiro, Hana Inoue, Masato Konishi

**Affiliations:** Department of Physiology, Tokyo Medical University, Tokyo, Japan; The University of Manchester, United Kingdom

## Abstract

To study possible modulation of Mg^2+^ transport in low Mg^2+^ conditions, we fed either a Mg-deficient diet or a Mg-containing diet (control) to Wistar rats for 1–6 weeks. Total Mg concentrations in serum and cardiac ventricular tissues were measured by atomic absorption spectroscopy. Intracellular free Mg^2+^ concentration ([Mg^2+^]_i_) of ventricular myocytes was measured with the fluorescent indicator furaptra. Mg^2+^ transport rates, rates of Mg^2+^ influx and Mg^2+^ efflux, were estimated from the rates of change in [Mg^2+^]_i_ during Mg loading/depletion and recovery procedures. In Mg-deficient rats, the serum total Mg concentration (0.29±0.026 mM) was significantly lower than in control rats (0.86±0.072 mM) after 4–6 weeks of Mg deficiency. However, neither total Mg concentration in ventricular tissues nor [Mg^2+^]_i_ of ventricular myocytes was significantly different between Mg-deficient rats and control rats. The rates of Mg^2+^ influx and efflux were not significantly different in both groups. In addition, quantitative RT-PCR revealed that Mg deficiency did not substantially change mRNA expression levels of known Mg^2+^ channels/transporters (TRPM6, TRPM7, MagT1, SLC41A1 and ACDP2) in heart and kidney tissues. These results suggest that [Mg^2+^]_i_ as well as the total Mg content of cardiac myocytes, was well maintained even under chronic hypomagnesemia without persistent modulation in function and expression of major Mg^2+^ channels/transporters in the heart.

## Introduction

Intracellular Mg^2+^ plays crucial roles in cellular functions, including DNA synthesis, enzyme activities, and gating of ion channels. In cardiac myocytes, Mg^2+^ regulates Ca^2+^ and K^+^ channels [1]–[3], local Ca^2+^ release from sarcoplasmic reticulum [4] and Ca^2+^ sensitivity of intracellular buffer sites [5]. Abnormalities in cellular Mg^2+^ homeostasis might cause cardiovascular diseases, such as arrhythmias and heart failure.

Regarding the physiological condition of rat ventricular myocytes, the intracellular free Mg^2+^ concentration ([Mg^2+^]_i_) is in the range of 0.8–1.0 mM [6], and it is thought to be regulated by the balance between passive influx driven by the electrochemical gradient of ion and active extrusion in exchange for Na^+^ influx (i.e., putative Na^+^/Mg^2+^ exchange). We have reported that Mg^2+^ efflux through Na^+^/Mg^2+^ exchange is activated by a slight increase in [Mg^2+^]_i_ [7].

In the last decade, several Mg^2+^ channels/transporters have been identified in eukaryotes. Among them, the melastatin subfamily 6 and 7 of the transient receptor potential cation channels (TRPM6 and 7, respectively) [8]–[11], MagT1 [12], SLC41A1 [13] and ACDP2 [14] are suggested to be the Mg^2+^ channels/transporters implicated in Mg^2+^ homeostasis of mammalian cells. It has been reported that the function and expression of such Mg^2+^ channels/transporters are modified by extracellular and intracellular levels of Mg^2+^. In mammalian epithelial cells (HC11), low [Mg^2+^]_i_ and high [Mg^2+^]_i_ accelerated, respectively, Mg^2+^ influx and efflux. When the cells were incubated in Mg^2+^-deprived medium, TRPM6 mRNA and protein levels were upregulated [15]. TRPM6 protein expressions in breast and kidney tissues were modulated by dietary Mg^2+^, whereas TRPM7 expression remained unaltered [16].

In this study, we fed rats a Mg-deficient diet, and examined changes in Mg^2+^ transport functions and related gene expressions in cardiac myocytes. We unexpectedly found that neither Mg^2+^ transport rates nor mRNA expressions of major Mg^2+^ channels/transporters were significantly altered in rats fed a Mg-deficient diet for 4–6 weeks, in spite of severe hypomagnesemia. Portions of this work have been reported in abstract form [17].

## Materials and Methods

### Animals and Diets

All experimental procedures involving animals were approved in advance by the institutional Animal Care and Use Committee of Tokyo Medical University (Permit Number: S-23013), and were performed in accordance with the “Guidelines for Proper Conduct of Animal Experiments” approved by the Science Council of Japan.

Male Wistar rats (8 weeks old, unless otherwise stated) were fed either a control diet (AIN93M diet that contained 0.05% magnesium [18]) with tap water or a Mg-deficient diet with distilled water. Food and water were freely available. The Mg-deficient diet was made by removal of MgO from AIN93M. The control diet and the Mg-deficient diet were purchased from Oriental Yeast Co., Ltd. (Tokyo).

Each rat was deeply anesthetized by intraperitoneal injection of pentobarbital (100–120 mg/kg bw). After chest opening, a blood sample (3–5 ml) was collected from the left ventricular cavity by puncture, and the heart was quickly excised. Blood samples and heart ventricles were immediately processed for atomic absorption spectroscopy (AAS) to analyze mineral concentrations. For isolation of ventricular myocytes, the aorta of the excised heart was cannulated for Langendorff perfusion and subsequent enzymatic dispersion of single cells [19].

### Measurements of Total Mineral Concentrations

Total mineral concentrations in serum and tissues were measured by AAS. Serum was treated with 1N nitric acid (HNO_3_) and 20% trichloroacetic acid (TCA) to deproteinize. After centrifugal separation, the supernatant was diluted with 0.4N HNO_3_ and 8% TCA to determine concentrations of Mg, Ca, Na and K using a Spectra 880 atomic absorption spectrometer (Varian Inc., Palo Alto, CA, USA). Mg and Ca contents of ventricular tissues were determined by AAS after wet-digestion of ventricles with trace element-grade HNO_3_ and hydrogen peroxide (Wako Chemicals, Osaka) and appropriate dilution with 0.1N hydrochloric acid.

### Measurements of [Mg^2+^]_i_ with the Fluorescent Mg^2+^ Indicator Furaptra

The instruments and procedures for the measurements of fluorescence signals from single myocytes have been described previously [7], [20]. In brief, single ventricular myocytes enzymatically dissociated from rat hearts [19] were placed in a chamber on the stage of an inverted microscope (TE300; Nikon, Tokyo) and were superfused with normal Tyrode’s solution containing (mM): 135 NaCl, 5.4 KCl, 1.0 CaCl_2_, 1.0 MgCl_2_, 0.33 NaH_2_PO_4_, 5.0 glucose and 10 HEPES (pH 7.40 at 25°C by NaOH). After the measurement of background fluorescence and indicator loading by incubation with 5 µM furaptra AM (mag-fura-2 AM; Invitrogen, Carlsbad, CA, USA) in normal Tyrode’s solution for 15 min at room temperature, the AM ester was washed out with Ca^2+^-free Tyrode’s solution that contained 0.1 mM K_2_EGTA in place of 1.0 mM CaCl_2_ of normal Tyrode’s solution (Table 1) for at least 10 min.

**Table 1:** Major constituents of the bathing solutions.

(mM)	NaCl	NMDG	KCl	MgCl_2_	MgMs_2_		[Mg^2+^]	[Na^+^]	[K^+^]
Ca^2+^-free Tyrode’s	135		5.4	1.0	0		1	140	5.4
Mg-loading	0	101	5.4	17.9	6		24	1.6	5.4
Mg-depleting	0	0	140	0	0		0	0.3	145
Mg-free NMDG	0	135	5.4	0	0		0	0.3	5.4

Ms, methanesulfonate; NMDG, n-methyl-D-glutamine. All solutions contained 0.1 mM K_2_EGTA, 0.33 mM NaH_2_PO_4_ and 10 mM HEPES, and were essentially free of Ca^2+^, and had osmolality of ∼290 mOsm/kg H_2_O. The pH of the solutions was adjusted to 7.40 with NaOH (for Ca^2+^-free Tyrode’s solution), with NaOH plus HCl (for Mg-loading solution), with KOH (for Mg-depleting solution) or HCl (for Mg-free NMDG solution). Final concentrations of Mg^2+^, Na^+^ and K^+^ are shown in the rightmost three columns.

All fluorescence measurements were carried out at 25°C, except during Mg^2+^ depletion (see below), because *in vivo* parameters for calibration of furaptra fluorescence (in terms of [Mg^2+^]_i_) have been determined at 25°C [6]. The intracellular furaptra was alternately excited with 350 nm and 382 nm light beams at 10 ms intervals, and the fluorescence at 500 nm (25 nm bandwidth) was detected from the entire volume of single cells. At each excitation wavelength, the background fluorescence measured for each cell before indicator loading was subtracted from the total fluorescence measured after indicator loading to yield indicator fluorescence intensity. The ratio of furaptra fluorescence intensities excited at 382 nm and 350 nm [R = F(382)/F(350)] was converted to [Mg^2+^]_i_ according to the equation:
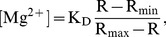
(1)where K_D_ is the dissociation constant, and R_min_ and R_max_ are R values at zero [Mg^2+^] and saturating [Mg^2+^], respectively. We used the parameter values previously estimated in rat ventricular myocytes at 25°C: K_D_ = 5.30 mM, R_min_ = 0.969, and R_max_ = 0.223 [6].

### Analyses of Mg^2+^ Influx rates

As we measured the changes in [Mg^2+^]_i_ caused by net flux (sum of influx and efflux) of Mg^2+^ across the cell membrane, experimental protocols were designed to minimize Mg^2+^ efflux for Mg^2+^ influx measurements (or to minimize Mg^2+^ influx for Mg^2+^ efflux measurements). We used the following two protocols to evaluate the rates of Mg^2+^ influx.

#### 1) Mg^2+^ loading

The myocytes were loaded with Mg^2+^ by incubation in a Mg-loading solution that contained 24 mM Mg^2+^ (Table 1) at ∼25°C. The Mg-loading solution contained a very low [Na^+^] to inhibit the Na^+^-dependent Mg^2+^ efflux, the major pathway for Mg^2+^ efflux in cardiac myocytes [20]. Average rates of rise in [Mg^2+^]_i_ for 3 h were compared between the two diet groups (control diet and Mg-deficient diet).

#### 2) Mg^2+^ recovery after depletion

The myocytes were depleted of Mg^2+^ by incubation in the Mg-depleting solution (Table 1) for 20 min at 35°C, which caused a decrease in [Mg^2+^]_i_ from the basal level (∼0.9 mM) to 0.2–0.5 mM. In pilot experiments the rate of decrease in [Mg^2+^]_ i_ was significantly higher at 35°C than at 25°C, thus we chose 35°C (rather than 25°C) to minimize possible cell damage caused by prolonged exposure to the high-K solution.

**Figure 1:**
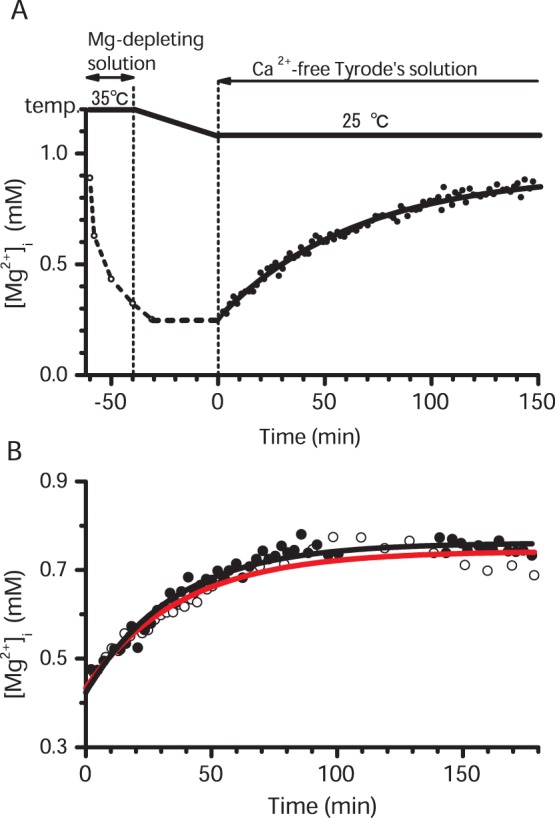
Observation of Mg^2+^ influx in the cells depleted of Mg^2+^. (A) Experimental protocol for depletion and recovery of Mg^2+^. A cell was depleted of Mg^2+^ by incubation in the Mg^2+^-depletion solution at 35°C for 20 min. During this period, [Mg^2+^]_i_ decreased from the basal level (∼0.9 mM) to the lower levels of 0.2–0.5 mM (open circles and dotted lines). Subsequent application of Ca^2+^-free Tyrode’s solution that contained 1 mM Mg^2+^ (25°C) caused a recovery of [Mg^2+^]_i_ towards the basal level (filled circles). The course of the recovery was well fitted by a single exponential function, as indicated by the continuous line. (B) Examples of two experimental runs showing Mg^2+^ influx in the Mg^2+^-depleted cells isolated from a control rat (filled circles) or a Mg-deficient rat (open circles). For filled circles and open circles, sets of values between 0 and 180 min were least-squares fitted by the exponential function (continuous lines: black for filled circles, red for open circles)



The bathing solution was then switched to the Mg-free NMDG solution (Table 1) for 30–40 min, during which the solution temperature gradually returned to 25°C, while the lowered [Mg^2+^]_i_ level was maintained (see Fig. 1A). When the Mg^2+^-depleted myocytes were superfused with Ca^2+^-free Tyrode’s solution that contained normal levels of Na^+^, K^+^ and Mg^2+^ (at 25°C), [Mg^2+^]_i_ started to rise and reached a plateau in ∼2 h near the initial basal level (Fig. 1A). We followed [Mg^2+^]_i_ recovery at ∼2 min intervals for 150–180 min, and found that the time course of the recovery could be well fitted by a single exponential function of time (t),

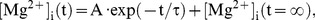
(2)where A is a constant and τ is a time constant (Fig. 1A). Because the [Mg^2+^]_i_ recovery is likely caused by the influx of Mg^2+^, the first derivative of the recovery function (2) is thought to reflect the rate of Mg^2+^ influx,



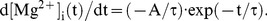
(3)We used the value of d[Mg^2+^]_i_ (t)/dt at time 0, −A/τ, as an index of the initial rate of Mg^2+^ influx. Note that initial [Mg^2+^]_i_ of Mg^2+^ recovery was 0.2–0.5 mM, at which the Na^+^-dependent Mg^2+^ efflux activity was thought to be negligible [7].

### Analyses of Mg^2+^ Efflux Rates

After the myocytes were loaded with Mg^2+^ in Mg-loading solution (Table 1) to reach [Mg^2+^]_i_≥1.5 mM, superfusion of Ca^2+^-free Tyrode’s solution that contained 140 mM Na^+^ (25°C) induced a rapid decrease of [Mg^2+^]_i_ [20]. The initial rate of decrease in [Mg^2+^]_i_ was estimated by linear regression of data points spanning 120 s after the addition of extracellular Na^+^, and it was considered to reflect the rate of Mg^2+^ efflux, as previously reported [7], [20]–[22]. In this condition (∼1.5 mM [Mg^2+^]_i_ and 1 mM [Mg^2+^]_o_), Mg^2+^ influx is thought to be negligible, because the Mg^2+^ influx rate measured in the absence of extracellular Na^+^ was much lower even under reversed Mg^2+^ gradient (1–1.5 mM [Mg^2+^]_i_ and 93 mM [Mg^2+^]_o_) [20].

### Quantitative Real-time PCR

Total RNA was extracted from segments of cardiac ventricles and kidneys using the SV total RNA Isolation System (Promega, Madison, WI, USA), following the manufacturer’s protocols. The heart and the kidney were quickly excised from anesthetized rats, and stored in RNA-later® solution (Ambion, Life Technologies, Carlsbad, CA, USA) until used. High Capacity RNA to cDNA kit (Applied Biosystems, Life Technologies) was used for reverse transcriptase reactions. The expression levels of TRPM6, TRPM7, MagT1, SLC41A1 and ACDP2 were determined by quantitative real-time PCR in each sample using ABI 7500 Real-Time PCR system (Life Technologies). The expression level of the house-keeping gene GAPDH was used as an internal control. Primers and probes used for target genes were TaqMan Gene Expression Assays (Rn99999916_s1 for GAPDH; Rn01760130_m1 for TRPM6; Rn00586779_m1 for TRPM7; Rn00588477_m1 for MagT1; Rn01484050_m1 for SLC41A1; Rn01410702_m1 for ACDP2) purchased from Applied Biosystems, Life Technologies.

### Data Analysis

Linear and nonlinear least-squares fittings were performed with the program Origin (Ver. 8.1, OriginLab, Northampton, MA, USA). Statistical values are expressed as the mean±SEM. Differences between groups were analyzed by Student’s two-tailed *t*-test (or 2-way ANOVA) with the significance level set at p<0.05.

## Results

### Mg-deficient Rats

**Figure 2:**
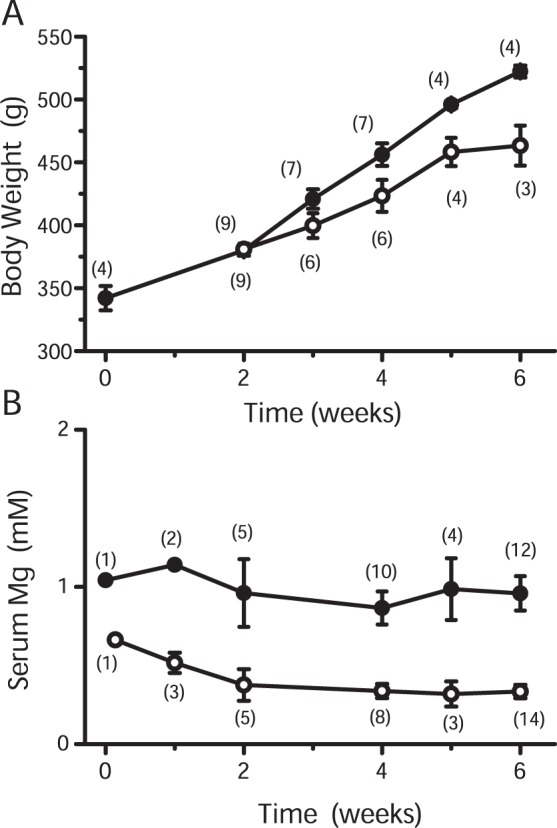
Body weight and serum Mg concentration during Mg-deficiency. Rats (8 weeks old) were divided into two groups and fed either a control diet or a Mg-deficient diet for 1 to 6 weeks. Body weight (A) and total concentrations of serum Mg (B) were plotted as a function of time after either the control diet (filled circles) or the Mg-deficient diet (open circles) was started at time 0. For serum Mg, the small number of data obtained from 4-week-old rats (at the beginning of feeding) was also included, because the serum Mg concentration was similar between the 8-week-old and 4-week-old rats in the pilot experiments. Each symbol represents mean±SEM of the number of rats indicated nearby. For both A and B, the average values of Mg-deficient rats were significantly different from those of control rats (p<0.01, 2-way ANOVA).

When rats were fed with the Mg-deficient diet for a prolonged period, their growth was retarded. Average body weight of the Mg-deficient diet group was significantly lower than that of the control diet group after feeding for 5 weeks or longer (Fig. 2A). Also, four out of 9 rats showed transient hyperemia during the second week of the diet, which disappeared at the third week or later (not shown). The retarded growth and early transient hyperemia are consistent with signs of Mg deficiency as reported earlier [23], [24].

The serum Mg concentration quickly decreased after starting the Mg-deficient diet, and was significantly lower than the control rats after 2 weeks of feeding (Fig. 2B). Thereafter the levels of serum Mg remained low until the end of the feeding period of 6 weeks (Fig. 2B). The results clearly confirmed that the rats fed the Mg-deficient diet for 2 weeks or longer are actually deficient in Mg, i.e., Mg-deficient rats. In contrast, in the rats fed the control diet the serum Mg concentration remained approximately constant at 0.9–1 mM throughout the feeding period (1–6 weeks) (Fig. 2B).

### Total Mineral Concentrations in Serum and Ventricular Tissues

In addition to Mg, we measured the serum concentrations of Ca, Na and K of rats fed the control diet and those receiving the Mg-deficient diet for 4–6 weeks (Table 2). In Mg-deficient rats, the average serum Mg concentration was about 1/3 of that in control rats, whereas Ca, Na and K levels were not significantly different from those in control rats (Table 2). We also measured the tissue contents of Mg and Ca in cardiac ventricles of rats fed the control diet and those receiving the Mg-deficient diet for 4–6 weeks, and found no significant difference in Mg and Ca contents between the two groups (Table 2), in spite of very low serum concentrations of Mg in the Mg-deficient rats.

**Table 2:** Total mineral concentrations in serum and ventricular tissues.

Serum concentration (mM)	Control		Mg-deficient	
Mg	0.86±0.072	(22)	0.29±0.026**	(20)
Ca	2.63±0.20	(10)	2.52±0.08	(6)
Na	138±5.4	(9)	150±11	(6)
K	3.43±0.13	(9)	3.03±0.14	(6)
**Tissue concentration (µM/g)**				
Mg	193.25±8.03	(4)	193.25±8.10	(4)
Ca	78.26±7.15	(4)	91.70±21.23	(4)

Rats (8 weeks old) were fed the control diet (Control) or the Mg-deficient diet (Mg-deficient) for 4–6 weeks, and total mineral concentrations were measured by AAS. Each data represents mean±SEM from the number of rats indicated in parentheses.

** p<0.01 (vs. control).

### Basal [Mg^2+^]_i_ of Ventricular Myocytes

Although the total Mg contents of ventricular tissues were not altered under serum Mg deficiency, redistribution of intracellular Mg could lead to changes in free Mg^2+^ concentration (i.e., [Mg^2+^]_i_). We therefore measured [Mg^2+^]_i_ of ventricular myocytes isolated from control rats and Mg-deficient rats, using the fluorescent indicator furaptra (see **Materials and Methods**). For myocytes incubated in Ca^2+^-free Tyrode’s solution that contained 1 mM Mg^2+^, basal levels of [Mg^2+^]_i_ were within the previously reported range [6], [7], [20]–[22] regardless of whether the myocytes were isolated from control rats or from Mg-deficient rats; average [Mg^2+^]_i_ values were not significantly different between the two groups (Table 3, 1 mM [Mg^2+^]_o_). However, because the perfusion solutions used for cell isolation and fluorescence measurements contained 1 mM Mg^2+^, one could argue that [Mg^2+^]_i_ of myocytes isolated from Mg-deficient rats might rise to the normal level during isolation and fluorescence measurement procedures. To rule out this possibility, we isolated the myocytes using solutions that contained only 0.2 mM Mg^2+^, and fluorescence measurements were carried out at 0.2 mM [Mg^2+^]_o_ (Mg^2+^ concentration of Ca^2+^-free Tyrode’s solution was reduced to 0.2 mM). [Mg^2+^]_i_ values thus obtained were still within the normal range, and were not significantly different between the two groups (Table 3, 0.2 mM [Mg^2+^]_o_). It should be also noted that [Mg^2+^]_i_ of myocytes isolated from Mg-deficient rats under the low Mg^2+^ condition (0.2 mM [Mg^2+^]_o_) was not significantly different from [Mg^2+^]_i_ of myocytes isolated from control rats under the normal Mg^2+^ condition (1 mM [Mg^2+^]_o_). Thus, it is likely that [Mg^2+^]_i_ of ventricular myocytes is sustained within the normal range even under the prolonged Mg deficiency for 4–6 weeks.

**Table 3:** Summary of results obtained from single ventricular myocytes.

Resting level of [Mg^2+^]_i_ (mM)	Control		Mg-deficient	
1 mM [Mg^2+^]_o_	0.916±0.015	(22)	0.947±0.016	(21)
0.2 mM [Mg^2+^]_o_	0.826±0.014	(6)	0.877±0.017	(6)
**Mg^2+^ influx rate (µM/s)**				
Mg-loading	0.061±0.0029	(22)	0.056±0.0034	(21)
Mg-recovery	0.27±0.04	(10)	0.22±0.05	(4)
**Mg^2+^ efflux rate (µM/s)**				
	−1.20±0.065	(5)	−1.04±0.057	(4)

**Figure 3:**
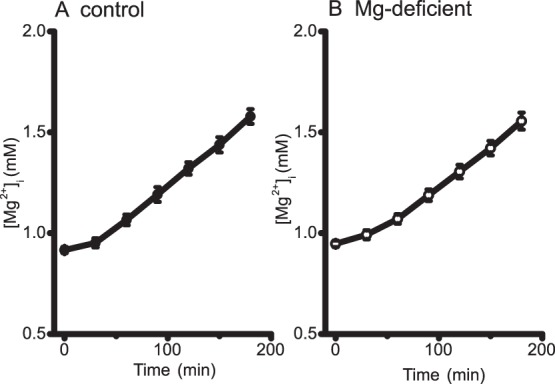
The increase in [Mg^2+^]_i_ by Mg^2+^-loading. Measurements of [Mg^2+^]_i_ from the cells isolated from control (A) or Mg-deficient (B) rats. The incubation solution of the cells was initially Ca^2+^-free Tyrode’s solution, and was switched to the Mg^2+^-loading solution at time 0 on the abscissa. Each symbol represents mean±SEM from 22 cells for (A) and 21 cells for (B).

**Figure 4:**
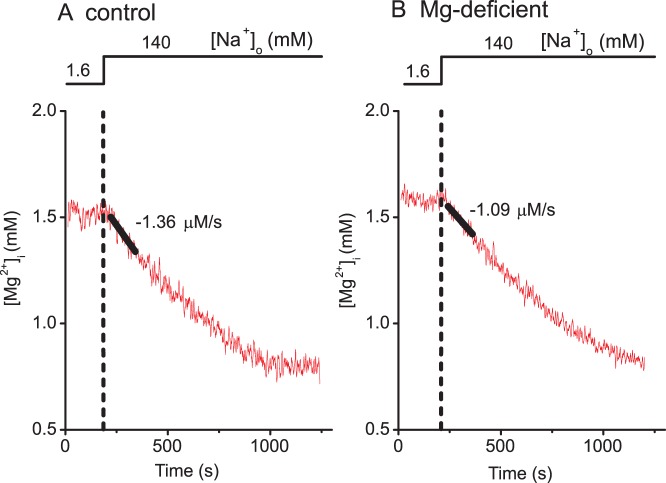
Measurements of extracellular Na^+^-dependent Mg^2+^ efflux. Cells isolated from control (A) or Mg-deficient (B) rats were initially incubated in Mg-loading solution that contained 1.6 mM Na^+^ and 24 mM Mg^2+^ (Table 1) for 3 h to load the cells with Mg^2+^. Then Ca^2+^-free Tyrode’s solution that contained 140 mM Na^+^ and 1 mM Mg^2+^ was introduced, as shown at the top. For each data set, a solid line was drawn by the linear least-squares fit to data points between 30 s and 150 s after solution exchange, and the initial rate of change in [Mg^2+^]_i_ estimated from the slope is indicated near the trace.

Rats (8 weeks old) were fed the control diet or the Mg-deficient diet for 4–6 weeks, and [Mg^2+^]_i_ was measured with the fluorescent indicator furaptra in the myocytes isolated from control rats (Control) and Mg-deficient rats (Mg-deficient). Each data represents mean ± SEM from the number of cells indicated in parentheses. The basal level of [Mg^2+^]_i_ was measured either at 1 mM [Mg^2+^]_o_ (i.e., Ca^2+^-free Tyrode’s solution) or at 0.2 mM [Mg^2+^]_o_ (Mg^2+^ concentration of Ca^2+^-free Tyrode’s solution was reduced to 0.2 mM). Mg^2+^ influx rates were estimated by two different methods: the rates of Mg^2+^ loading (as shown in Fig. 3) and the rates of Mg^2+^ recovery after depletion (as shown in Fig. 1). The Mg^2+^ efflux rate was estimated from the initial rate of decrease in [Mg^2+^]_i_ in the Mg^2+^-loaded cells as shown in Fig. 4. There was no significant difference between values obtained from control rats and those obtained from Mg-deficient rats.

Overall, the results suggest that Mg deficiency significantly alters neither total concentration nor free concentration of intracellular Mg in cardiac ventricles, in spite of the marked reduction of serum Mg by 66% (Table 2). This apparent discrepancy could be explained, if Mg^2+^ influx via the cell membrane is facilitated and/or Mg^2+^ efflux is suppressed under serum Mg deficiency. We therefore examined the activities of Mg^2+^ transport across the cell membrane and the expression levels of known Mg^2+^ channels/transporters in the following sections.

### Mg^2+^ Influx via Cell Membrane

#### 1) Mg^2+^ loading

After incubation of the myocytes in Mg-loading solution that contained 24 mM Mg^2+^ (Table 1), [Mg^2+^]_i_ was raised gradually and quasi-linearly from the basal levels of 0.9–1.0 mM to higher levels, and reached 1.5–1.9 mM in 3 h (Fig. 3). Because this increase in [Mg^2+^]_i_ is probably due to Mg loading of cells by the influx of Mg^2+^ from the extracellular space, the rate of rise of [Mg^2+^]_i_ is thought to reflect the Mg^2+^ influx rate. We measured [Mg^2+^]_i_ of the myocytes every 30 min during the Mg loading period of 3 h (Fig. 3), and estimated the average rate of rise in [Mg^2+^]_i_ for each cell. The rates of rise in [Mg^2+^]_i_ were, on average, 0.056±0.0034 µM/s and 0.061±0.0029 µM/s for the myocytes isolated from the Mg-deficient rats and the control rats, respectively (Table 3, Mg-loading). The average values were not significantly different between the two groups.

#### 2) Mg^2+^ recovery after depletion

During the 20-min exposure to the Mg-depleting solution (Table 1), [Mg^2+^]_i_ decreased, on average, by 0.48±0.054 mM (n = 4) and 0.57±0.038 mM (n = 10) in the cells isolated from, respectively, the Mg-deficient rats and the control rats. The decrements of [Mg^2+^]_i_ were not significantly different between the two groups.

After the [Mg^2+^]_i_ was lowered by incubation of the myocytes in Mg^2+^-free solutions (Table 1), the addition of 1 mM extracellular Mg^2+^ induced an immediate onset of a rise in [Mg^2+^]_i_ that reached a plateau near the basal level (Fig. 1A). Figure 1B compares [Mg^2+^]_i_ measurements (symbols) and fitted curves (solid lines) obtained from two myocytes (see **Materials and Methods**): one isolated from a Mg-deficient rat (open circles and a red line) and the other isolated from a control rat (filled circles and a black line). Note that the time courses of the [Mg^2+^]_i_ recovery were nearly superposable in these myocytes. The average values of the influx rate were 0.22±0.05 µM/s and 0.27±0.04 µM/s for the myocytes isolated from the Mg-deficient rats and the control rats, respectively (Table 3, Mg-recovery); these values were not significantly different. Thus, the results demonstrate little changes in Mg^2+^ influx activities even after prolonged Mg deficiency.

### Mg^2+^ Efflux via Cell Membrane

It has been demonstrated that Mg^2+^ efflux is critically dependent upon extracellular Na^+^ in cardiac myocytes [20], [25], [26]. In ventricular myocytes loaded with Mg^2+^, [Mg^2+^]_i_ remains elevated in the absence of extracellular Na^+^ (i.e., little or no efflux activity), but the addition of extracellular Na^+^ induces a rapid decrease in [Mg^2+^]_i_ (i.e., Na^+^-dependent Mg^2+^ efflux). The functional characteristics of the Na^+^-dependent Mg^2+^ efflux are consistent with the Na^+^/Mg^2+^ exchange which extrudes Mg^2+^ in exchange for Na^+^ influx [21], [22].

We evaluated the Na^+^-dependent Mg^2+^ efflux activity, a major efflux pathway in ventricular myocytes. It should be noted that the Mg^2+^ efflux is negligible at the basal [Mg^2+^]_i_ (∼0.9 mM), but it is activated by higher [Mg^2+^]_i_ at half-maximal activation at 1.5 mM [7]. Because of this strong [Mg^2+^]_i_ dependence, comparisons of the rates of Mg^2+^ efflux should be made at comparable [Mg^2+^]_i_ levels. Figure 4 shows the [Mg^2+^]_i_ recordings in two myocytes isolated from control rats (A) and Mg-deficient rats (B). After Mg loading of these myocytes for 3 h (with the protocol shown in Fig. 3), [Mg^2+^]_i_ levels were elevated to ≥1.5 mM ([Mg^2+^]_i_ at time 0–180 s in Fig. 4A and B). Extracellular application of 140 mM Na^+^ induced Mg^2+^ efflux with similar initial rates of decrease in [Mg^2+^]_i_ in A and B, as indicated by solid lines and numbers near the traces. For pooled data obtained from repeated experiments, elevated [Mg^2+^]_i_ levels after Mg loading were not significantly different between myocytes isolated from control rats (1.57±0.038 mM, n = 5) and those from Mg-deficient rats (1.58±0.053 mM, n = 4), and the initial rates of decrease in [Mg^2+^]_i_ upon Na^+^ addition were also not significantly different between these two groups (Table 3, Mg^2+^ efflux).

### Expression of Mg^2+^ Channels/Transporters

**Figure 5:**
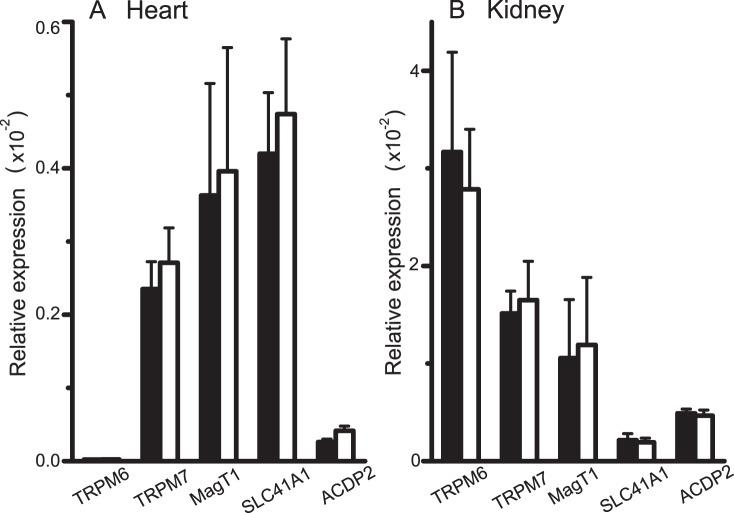
Expression of Mg^2+^ channels/transporters. Quantities of mRNA transcripts for TRPM6, TRPM7, MagT1, SLC41A1 and ACDP2 relative to that of GAPDH in ventricular tissue (A) and kidney tissue (B). Columns represent mean±SEMs of the data obtained from 6–7 rats (except for ACDP2, n = 3–4) fed the control diet (filled columns) and the Mg-deficient diet (open columns) for 4 weeks.

We measured and compared mRNA expression levels of known mammalian Mg^2+^ channels/transporters, TRPM6, TRPM7, MagT1, SLC41A1 and ACDP2, in cardiac ventricle and kidney tissues excised from control rats and Mg-deficient rats (Fig. 5). The TRPM6 transcripts were hardly detected in the heart, but were abundant in the kidney. Of these five channels/transporters, none of the relative quantities of transcripts were significantly different between control rats and Mg-deficient rats in the heart (A) and in the kidney (B). Thus, consistent with Mg^2+^ transport functions, Mg deficiency failed to induce significant changes in mRNA expression of major Mg^2+^ channels/transporters which are thought to play a vital role in cellular Mg homeostasis.

## Discussion

It has been reported that in mice fed a Mg-deficient diet chronic Mg deficiency does not significantly alter the Mg content of the heart tissue, in spite of severe hypomagnesemia [27]. However, because the Mg concentrations measured in this preceding study were not [Mg^2+^]_i_, but rather the total content of Mg, the authors did not exclude the possibility that [Mg^2+^]_i_ of cardiac myocytes might be reduced under Mg deficiency as a result of re-distribution of intracellular Mg, which caused a decrease in cardiac tolerance to hypoxia. The present study extends the earlier findings by 1) measurements of [Mg^2+^]_i_, 2) functional assays of Mg^2+^ influx and efflux rates, and 3) quantification of mRNA expression of known Mg^2+^ channels/transporters. We found that none of these measurements showed a significant alteration in rats fed a Mg-deficient diet for 4–6 weeks, although the serum levels of Mg fell to 1/3 in comparison with control rats fed a Mg-containing diet. These results suggest that [Mg^2+^]_i_ as well as total Mg content of cardiac myocytes is tightly regulated, under altered driving force for Mg^2+^ across the cell membrane, without persistent modulation of functions and expressions of major Mg^2+^ channels/transporters.

Although we cannot exclude the possibility that Mg^2+^ transport functions were influenced during cell isolation and manipulation procedures, it is unlikely that exposure of cells to high [Mg^2+^]_o_ (during Mg-loading) or low [Mg^2+^]_o_ (during Mg-depletion) overrides changes in cell membrane expression of channel/transporter proteins (i.e., insertion/removal from the cell membrane) previously induced by Mg deficiency *in vivo* based on the following findings: 1) Even at the very beginning of the Mg-loading, time courses of the rise in [Mg^2+^]_i_ (i.e., Mg^2+^ influx) were similar between the cells isolated from the Mg-deficient rats and those isolated from the control rats (Fig. 3). 2) The decrements of [Mg^2+^]_i_ (i.e., Mg^2+^ efflux) in the initial 20 min of Mg-depletion were not significantly different between the two groups (see **Results**).

Goytain and Quamme [14], [28], [29] reported significant increases in mRNA expressions of MagT1 and ACDP2 (in kidney), as well as SLC41A1 (in heart and kidney), when mice were fed a Mg-deficient diet for 5 days. The apparent discrepancy between these and the present results could be due to species differences (mice vs. rats), and the different times of Mg deficiency (5 days in Goytain and Quamme vs. 4 weeks in the present study). It is possible that Mg^2+^ channels/transporters are transiently upregulated by the onset of hypomagnesemia, and are normalized in chronic hypomagnesemia. On the other hand, prolongation of Mg-deficiency for a very long period (>6 weeks) also could significantly affect cellular Mg^2+^ handling. It is therefore important in future studies to follow the time courses of changes in function and expression of Mg^2+^ channels/transporters, as well as intracellular magnesium concentrations (free and total), during Mg deficiency.

[Mg^2+^]_i_ is maintained by the balance between Mg^2+^ influx and Mg^2+^ efflux. As a Mg^2+^ efflux pathway, we have studied active Mg^2+^ transport in exchange with Na^+^ influx (i.e., Na^+^/Mg^2+^ exchange) in rat ventricular myocytes [7], [20]–[22], [26], [30]. Because removal of extracellular Na^+^ nearly completely abolishes Mg^2+^ efflux activity [20], the Na^+^/Mg^2+^ exchange is thought to be the major pathway for Mg^2+^ efflux in cardiac myocytes. The Mg^2+^ transport rate by the Na^+^/Mg^2+^ exchange is critically dependent upon [Mg^2+^]_i_; the transport rate is almost null at resting [Mg^2+^]_i_, but a slight increase in [Mg^2+^]_i_ markedly activates the transport with half-maximal activation of ∼1.5 mM [7]. On the other hand, Mg^2+^ influx is thought to be passive via Mg^2+^ permeable channels (e.g., TRPM7 channels). It has been shown that conductance of TRPM7 channels increases with the reduction of [Mg^2+^]_i_ [8]. This [Mg^2+^]_i_ dependence of Mg^2+^ influx is also shown in Fig. 1. In Mg^2+^-depleted cells, [Mg^2+^]_i_ recovery is initially fast, but it slows as [Mg^2+^]_i_ approaches the basal level, reaching a plateau near the basal level (Fig. 1B). Overall, both Mg^2+^ influx and efflux pathways seem to be activated only when [Mg^2+^]_i_ deviates from the narrow range around its basal level. When serum Mg levels fall to 1/3 as observed in the present study, a transient decrease in [Mg^2+^]_i_ might occur. However, the decrease in [Mg^2+^]_i_ should turn off the Mg^2+^ efflux by the Na^+^/Mg^2+^ exchange mechanism. On the other hand, the Mg^2+^ influx should be activated, which increases [Mg^2+^]_i_ back to the basal level, albeit with a slower rate than that in normal [Mg^2+^]_o_. Thus, we suggest that [Mg^2+^]_i_ is maintained even under severe hypomagnesemia by [Mg^2+^]_i_-dependent regulation of Mg^2+^ influx and efflux pathways, although molecular mechanisms for such regulation are still unknown.

In studies with Mg-deficient animals, it is often assumed that a decrease in [Mg^2+^]_i_ caused by hypomagnesemia leads to changes in the function of cells and organs. The results of the present study clearly indicate that such a simple assumption cannot be applied at least to cardiac myocytes. It follows that heart dysfunctions observed in Mg-deficient animals are likely caused by low serum magnesium (i.e., total and free concentrations) and other accompanying changes, rather than by changes in [Mg^2+^]_i_.
